# Pancreatic Cancer Education: A Scoping Review of Evidence Across Patients, Professionals and the Public

**DOI:** 10.3390/curroncol33010033

**Published:** 2026-01-08

**Authors:** Olivia Watson, Gary Mitchell, Tara Anderson, Fadwa Al Halaiqa, Ahmad H. Abu Raddaha, Ashikin Atan, Susan McLaughlin, Stephanie Craig

**Affiliations:** 1 School of Nursing and Midwifery, Queen’s University Belfast, Belfast BT9 7BL, UK; gary.mitchell@qub.ac.uk (G.M.); tanderson@qub.ac.uk (T.A.); 2 Pre-Clinical Affairs, College of Nursing, Qatar University, Doha P.O. Box 2713, Qatar; f.alhalaiqa@qu.edu.qa (F.A.H.); a.aburaddaha@qu.edu.qa (A.H.A.R.); 3 Kulliyyah of Nursing, International Islamic University Malaysia (IIUM) Kuantan Campus, Kuantan 25200, Pahang Darul Makmur, Malaysia; ashikin_a@iium.edu.my; 4 Northern Ireland Pancreatic Cancer (NIPANC), 384 Belmont Road, Belfast BT4 2NF, UK

**Keywords:** pancreatic cancer, cancer, education, literature review, scoping review, cancer awareness, public health, patients, healthcare professionals, students

## Abstract

**Background**: Pancreatic cancer is the least survivable malignancy, with five-year survival below 10%. Its vague, non-specific symptoms contribute to late diagnosis and poor outcomes. Targeted education for healthcare professionals, students, patients, carers, and the public may improve awareness, confidence, and early help-seeking. This scoping review aimed to map and synthesize peer-reviewed evidence on pancreatic cancer education, identifying intervention types, outcomes, and gaps in knowledge. **Methods**: A scoping review was undertaken using the Joanna Briggs Institute (JBI) framework and the Arksey and O’Malley framework and reported in accordance with PRISMA-ScR guidelines. The protocol was registered on the Open Science Framework. Four databases (MEDLINE, Embase, CINAHL, PsycINFO) were searched for English-language, peer-reviewed studies evaluating educational interventions on pancreatic cancer for healthcare students, professionals, patients, carers, or the public. Grey literature was excluded to maintain a consistent methodological standard. Data were charted and synthesised narratively. **Results**: Nine studies (2018–2024) met inclusion criteria, predominantly from high-income countries. Interventions targeted students and professionals (*n* = 3), patients (*n* = 2), the public (*n* = 2), or mixed groups (*n* = 2), using modalities such as team-based learning, workshops, virtual reality, serious games, and digital animations. Four interrelated themes were identified, encompassing (1) Self-efficacy; (2) Knowledge; (3) Behavior; and (4) Acceptability. Digital and interactive approaches demonstrated particularly strong engagement and learning gains. **Conclusions**: Pancreatic cancer education shows clear potential to enhance knowledge, confidence, and engagement across diverse audiences. Digital platforms offer scalable opportunities but require quality assurance and long-term evaluation to sustain impact. The evidence base remains limited and fragmented, highlighting the need for validated outcome measures, longitudinal research, and greater international representation to support the integration of education into a global pancreatic cancer control strategy. Future studies should also evaluate how educational interventions influence clinical practice and real-world help-seeking behaviour.

## Simple Summary

Pancreatic cancer is one of the deadliest cancers because it is often diagnosed late, when treatment options are limited. Many patients, families, and healthcare professionals report uncertainty about symptoms, treatment options, and care pathways, highlighting the importance of effective education. This review examined existing research on educational interventions about pancreatic cancer for healthcare students, professionals, patients, carers, and the public. Nine studies were identified, using approaches such as workshops, digital animations, virtual reality, and online games. Most interventions improved knowledge, confidence, or engagement, particularly when interactive or digital methods were used. However, the evidence base is small, mostly from high-income countries, and rarely examines long-term impact. This review shows that pancreatic cancer education has promise but remains underdeveloped. Future research should focus on high-quality, well-evaluated educational programmes that support earlier symptom recognition, informed decision-making, and better care for people affected by pancreatic cancer.

## 1. Introduction

Globally, pancreatic cancer is recognised as one of the most lethal malignancies and a growing public health challenge [1]. Despite representing a smaller proportion of total cancer cases worldwide, it accounts for a disproportionately high number of cancer-related deaths, ranking seventh globally for cancer mortality [2]. Worldwide, five-year survival remains below 10%, with minimal variation between regions [3] these outcomes highlight the need for improved awareness and education across both professional and public levels. In the United Kingdom (UK), it is the fifth most common cause of cancer-related death, with the most recent national statistics in the UK showing that, every day, approximately 30 new cases of pancreatic cancer are diagnosed, and 26 deaths are recorded due to the disease [4]. The persistently poor prognosis of pancreatic cancer can, in large part, be attributed to challenges in early recognition arising from its vague and often non-specific symptomatology. Improving early recognition and strengthening awareness at both public and clinical levels are therefore critical priorities.

The clinical presentation of pancreatic cancer is often complex and vague, increasing the risk of symptoms being misdiagnosed or missed altogether. Common symptoms include jaundice, abdominal pain, unexplained weight loss and changes in bowel habits [5]. However, in the early stages, many individuals are asymptomatic or present with non-specific symptoms that are easily attributed to more common, benign conditions [6,7]. Strengthening early recognition pathways may therefore play a critical role in improving prognostic trajectories.

However, earlier presentations may also be met with barriers. Research highlights that patients frequently experience significant delays between the onset of symptoms and diagnosis, often due to repeated healthcare visits without referral for appropriate imaging or specialist review [8,9]. Therefore, strengthening the awareness and clinical questioning of front-line healthcare providers, particularly doctors, nurses, and pharmacists, combined with educating patients and carers to recognise symptoms and advocate for themselves more effectively, could improve early detection [10]. Providing robust, evidence-based educational resources enables early investigation and more appropriate referral, while empowering patients may reduce missed opportunities for timely care [9]. Improving consistency of information across the care pathway may also help minimize repeated consultations and diagnostic uncertainty.

This education is vital for all healthcare professionals, particularly those based in a primary care setting [11]. These patients were found to be 72% less likely to be eligible for active cancer treatment [7]. Over 40% of patients with pancreatic cancer reported visiting their general practitioner (GP) three or more times before being referred to specialist services within the UK [11,12]. This repeated consultation pattern points to the difficulty in distinguishing early pancreatic cancer from more common conditions but also emphasizes the potential value of targeted education to improve symptom recognition and prompt referral. Embedding focused pancreatic cancer content into primary care training could help clinicians identify red-flag symptoms earlier and refine referral decision-making.

Educational initiatives should not be limited to healthcare professionals alone. Raising public awareness of pancreatic cancer’s potential warning signs could encourage earlier presentation to healthcare services, particularly in individuals with recognised risk factors such as smoking, chronic pancreatitis, obesity, or family history of the disease [12]. For healthcare professionals, especially nurses, doctors, and pharmacists, enhanced education on pancreatic cancer presentation and referral criteria could help address one of the main bottlenecks in the diagnostic pathway. For the public, campaigns focusing on key symptoms, such as unexplained jaundice, persistent abdominal pain, or unexplained weight loss, could improve self-advocacy and demand for further investigation. Coordinated messaging across public and professional domains may further enhance diagnostic efficiency.

This scoping review (ScR) therefore aimed to synthesize the existing literature to map what is currently known about pancreatic cancer education. Previous potentially relevant reviews have focused on supportive care needs of those affected by pancreatic cancer [13], and shared decision-making among pancreatic cancer patients [14]. These reviews highlight the need for information and awareness amongst patient and carers groups, and healthcare professionals. This aims to address these gaps in existing literature. Accordingly, this review aims to synthesize existing evidence to identify educational strategies, evaluate their effectiveness, and highlight gaps in pancreatic cancer education. The scoping review methodology was chosen because it allows for a comprehensive mapping of available evidence and can identify knowledge gaps that may not be apparent through a more narrowly focused systematic review [15]. In addition, a scoping review allows for synthesis of a heterogeneous evidence base to include a diverse range of educational approaches, study designs, outcomes, and population members. This approach provides a broad foundation for future intervention development, implementation, and evaluation in pancreatic cancer education.

## 2. Materials and Methods

### 2.1. Aim and Objectives

The scoping review aimed to map and characterize the peer-reviewed evidence on pancreatic cancer education for healthcare students, healthcare professionals, patients, carers, and the public. The objectives were to: (1) identify the range of educational interventions and resources addressing pancreatic cancer; (2) describe their purposes, contents, delivery modes and intended learning outcomes; (3) map outcomes on knowledge, self-efficacy, behaviour change and educational acceptability; and (4) highlight gaps to inform future education and research. These clearly defined objectives were intended to enhance methodological transparency and ensure alignment with scoping review guidance.

### 2.2. Design

A scoping review was conducted following the Joanna Briggs Institute (JBI) methodology for scoping reviews [16] which builds upon Arksey and O’Malley’s earlier framework [17] and is reported in line with the Preferred Reporting Items for Systematic reviews and Meta-Analyses extension for Scoping Reviews (PRISMA-ScR) checklist [18]. The completed checklist is available in Supplementary Table S1. The review protocol was registered on the Open Science Framework (OSF) on 3 June 2025. The initial database searches were conducted in April 2025, with protocol registration occurring prior to screening and data extraction. The protocol was not amended following registration (registration DOI: https://osf.io/ynkbf/Accessed 31 May 2025). Registering the protocol strengthened methodological rigour and minimized the risk of reporting bias.

### 2.3. Sources of Evidence

Experimental and quasi-experimental studies (randomized or non-randomized controlled trials, pre-post studies, interrupted time series), analytical observational studies (cohort, case–control, or analytical cross-sectional) that evaluated educational impact, and qualitative research exploring experience or acceptability (e.g., phenomenology, grounded theory, qualitative description) were all considered for inclusion. Systematic reviews containing extractable education-focused data were also considered, and reference lists of the included studies were screened. Purely descriptive or opinion-based papers were excluded unless they evaluated a specific educational intervention with measurable outcomes. This structured approach ensured that only methodologically meaningful educational evaluations were considered.

Only English-language, peer-reviewed publications were included. Grey literature (e.g., reports, conference abstracts, dissertations, and unpublished resources) was excluded. While scoping review guidance does not mandate inclusion of grey literature, its inclusion should be aligned with the review objectives. This review aimed to map and evaluate educational interventions with explicitly reported methods and measurable outcomes. Preliminary scoping indicated that grey literature in this field largely consisted of descriptive resources or awareness campaigns without formal evaluation or transparent methodology. Excluding grey literature therefore enhanced methodological consistency, reproducibility, and interpretability of findings, while ensuring that included studies allowed meaningful comparison of educational outcomes. Additional eligibility criteria were formulated in accordance with the population, concept, context (PCC) framework as recommended by the JBI guidance [16] and are summarized in Table 1. Using the PCC framework provided a coherent structure for defining study relevance and scope.

### 2.4. Search Strategy

The review team searched four electronic databases (MEDLINE, Embase, CINAHL and PsycINFO) in April 2025 and re-ran these searches in November 2025 for published studies, with no date limits and English-language restriction. All records retrieved from the November update were screened and fully incorporated into the final study selection, results, and PRISMA-ScR flow diagram. Terms were adapted and indexed for each database. In accordance with PRISMA-ScR recommendations, the full search strategy for Embase is provided in Table 2 to enhance transparency and reproducibility. An initial scoping search of Google Scholar informed the final strategy by harvesting title/abstract terms and indexing of relevant records.

### 2.5. Data Charting (Extraction)

Covidence software (https://www.covidence.org/ Accessed 1 May 2025) was used to aid in the removal of duplicates and the screening of the literature. A structured data-charting form drawing on JBI templates was developed and tailored to accommodate quantitative, qualitative and mixed-method designs [18]. Two reviewers (OW and SC) independently piloted the form on a subset of records to ensure consistency and capture of all pre-specified items; minor refinements were made (e.g., separating ‘intervention delivery’ from ‘learning activities’ fields). All included studies were then charted and entries verified via cross-checking. Disagreements were resolved by discussion or arbitration (GM). Extracted items included: bibliographic details; country and setting; target population; study design; sampling/participants; intervention aim and theoretical basis (if stated); content and com-ponents; duration/intensity; delivery modality and educator; comparators (if any); out-come measures (knowledge/awareness/attitudes/self-efficacy/behaviour); timing of assessment; analytic approach; key findings; and authors’ stated limitations (Supplementary Table S2). The data were tabulated to support mapping across populations, intervention types and outcomes [19,20].

### 2.6. Synthesis of Results

Findings were synthesised using a narrative approach, following the methodological guidance of Popay and colleagues to enhance transparency and reproducibility [21]. Thematic synthesis was employed where data heterogeneity precluded meta-analysis. The synthesis was undertaken in four interlinked stages. First, a conceptual model was developed articulating a preliminary framework describing how educational interventions might influence awareness, knowledge, attitudes, and self-efficacy across different populations through their content, pedagogical approach, and delivery mechanisms. This model supported a structured, theory-aligned interpretation of findings. Next, an initial synthesis was constructed by grouping studies according to the target population (students, health professionals, patients or carers, and members of the public) and intervention type (e.g., didactic teaching, e-learning, simulation-based education, written materials, and multi-component programmes). This stage involved a detailed textual description and tabulation to organise the evidence.

Following this, analytical themes were derived inductively and iteratively by examining patterns, similarities, and differences within and across these groups [22]. Descriptive codes were first assigned to key findings reported in each study (e.g., improved knowledge, enhanced communication confidence, increased patient preparedness). These codes were then compared and clustered to form higher-order categories, which were refined through repeated reading and constant comparison to identify overarching themes that captured how educational interventions influenced outcomes [23]. Relationships within and between studies were explored by mapping these emerging themes to intervention features and contextual factors such as setting, duration, and educator role. An iterative approach enhanced depth and coherence across heterogeneous study designs.

Finally, the robustness of the synthesis was assessed by considering methodological quality, completeness of data, and the consistency of reported effects when drawing conclusions. Given the substantial heterogeneity in populations, interventions, comparators, and outcomes, statistical pooling and meta-analysis were not appropriate, this aligns with JBI recommendations for scoping reviews which focus on mapping breadth rather than quantifying effects. Instead, a narrative synthesis was adopted to capture the breadth and diversity of the evidence base. To aid clarity and communication, findings were presented using structured evidence tables alongside a descriptive thematic narrative, consistent with PRISMA-ScR guidance [16].

## 3. Results

### 3.1. Search Results

Search yields by source were as follows: Embase (*n* = 4641), MEDLINE (*n* = 2661), CINAHL (*n* = 635), and PsycINFO (*n* = 58), totaling 8464 records before duplicates were removed.

**Figure 1:**
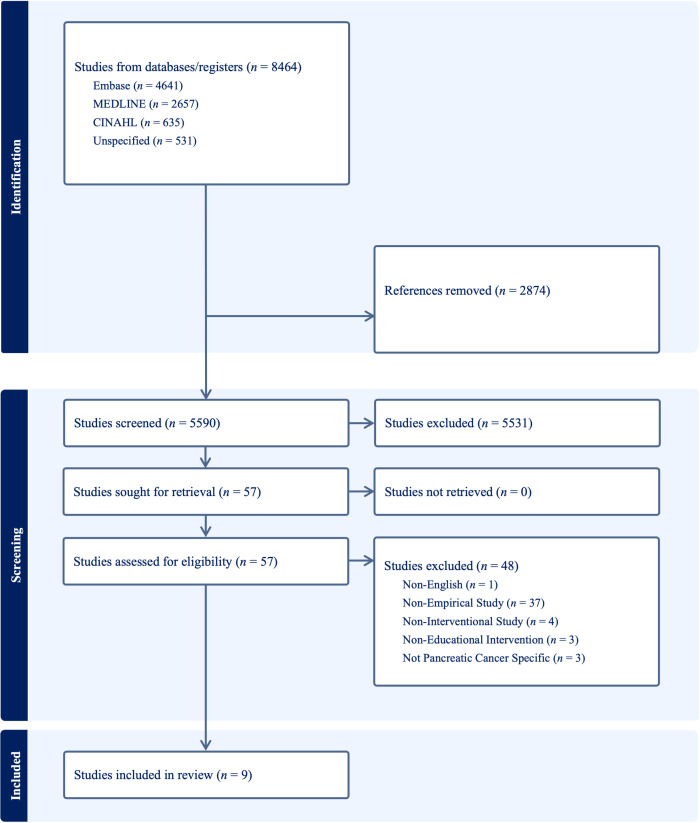
PRISMA-ScR flow diagram.

Automated tools for inclusion/exclusion beyond deduplication were not used. After removal of 2874 duplicates, 5590 records underwent title/abstract screening (Stage 1). Stage 1 screening was conducted independently and using a blinded approach by two reviewers (OW and SC), with 100% of records dual-screened. Conflicts were resolved by a third reviewer (GM). The same process was applied at full-text screening (Stage 2). Of 56 full texts assessed for eligibility, 9 met the inclusion criteria. Reasons for full-text exclusion (*n* = 48) were: non-English (*n* = 1), non-empirical study (*n* = 37), non-interventional study (*n* = 4), not an educational intervention (*n* = 3), and not pancreatic-specific outcomes (*n* = 3). No studies were unretrievable. The study selection process is presented in a PRISMA-ScR flow diagram in Figure 1 [17]. The November 2025 search update did not identify any additional eligible studies beyond those already included.

### 3.2. Critical Appraisal of Included Studies

In line with JBI guidance [18] that critical appraisal is optional but may be conducted to comment on the certainty or trustworthiness of a mapped evidence base, quality appraisal was undertaken within this review for context and interpretation (Table 3). The appropriate JBI Critical Appraisal Checklists were applied according to study design (e.g., RCTs, quasi-experimental, cohort, cross-sectional, qualitative) [18]. For mixed methods studies, the Mixed Methods Appraisal Tool was used to appraise integration and component quality [24]. Two reviewers (OW and SC) independently appraised each study with disagreements resolved by GM. Appraisal findings were not used to include/exclude studies but were considered when interpreting the body of evidence (e.g., risk of bias typical of pre–post designs, sampling limitations, validity of outcome measures). Of the nine included studies, five were appraised as moderate in quality, three as moderate–high, and one as high in quality. Most studies demonstrated clear aims, appropriate analyses, and strong alignment with learning objectives, though common limitations included the absence of control groups, reliance on self-reported data, and limited generalizability. Overall, the evidence base was considered methodologically adequate, with generally moderate to high quality across study designs. These findings are summarized below in Table 3. Conducting this appraisal provided valuable context for interpreting the robustness of the evidence base. Although appraisal outcomes did not inform study inclusion, they were used to contextualise interpretation of findings. Conclusions were weighted toward studies with stronger designs, larger samples, and validated measures, while findings from single-arm or self-reported studies were interpreted cautiously.

### 3.3. Study Characteristics

Nine studies published between 2018 and 2024 met the inclusion criteria. Most originated from the United States (*n* = 5) [26,27,28,29,30], with others from the United Kingdom (*n* = 2) [25,32], Germany (*n* = 1) [33], and a joint UK–Ireland study (*n* = 1) [32], indicating that pancreatic cancer education research remains largely concentrated in high-income settings. The limited global representation highlights an important equity gap in educational research.

Target audiences were diverse, encompassing healthcare students and professionals, patients and carers, and members of the public. Educational approaches ranged from traditional teaching methods (*n* = 2) [26,32], such as team-based learning (*n* = 1) [26] and professional workshops, to digital innovations including web-based serious games (*n* = 3) [25,29,30], virtual reality modules (*n* = 1) [33], multimedia resources (*n* = 1) [28], and online patient tools (*n* = 2) [27,31]. Collectively, these modalities were designed to address the four key outcome themes of self-efficacy, knowledge, behaviour and acceptability. This diversity reflects the breadth of educational modalities being explored but also highlights the heterogeneity of the field.

Study scale and design varied considerably, from 50 [31] to 1215 [32] participants. Larger studies included national or multi-site evaluations, while smaller studies focused on local contexts. Methodologies comprised randomised (*n* = 1) [28] or quasi-experimental trials (*n* = 3) [25,32,33], pre/post educational evaluations (*n* = 2) [26,32], cross-sectional surveys (*n* = 2) [27,31], content analysis (*n* = 1) [29], and retrospective analytics (*n* = 1) [30], with few employing control groups or longitudinal follow-up.

Outcomes most frequently assessed were knowledge and awareness (*n* = 6) [25,26,28,30,32,33], followed by self-efficacy (*n* = 3) [25,30,33] and satisfaction (*n* = 4) [25,28,30,33], mainly via pre/post self-report questionnaires. The lack of validated tools limits comparability across studies and reduces confidence in effect estimates. Qualitative data such as open-ended feedback were sometimes included to enrich interpretation.

Overall, the evidence demonstrates a growing but fragmented field, with interventions spanning diverse populations and modalities but limited by small sample sizes, heterogeneity, and methodological inconsistency. This highlights the need for more coherent, theory-informed approaches to pancreatic cancer education research.

### 3.4. Study Results

Where quantitative results are reported, effect sizes and statistical estimates are presented as reported by the original study authors. No additional statistical analyses were undertaken for this review. Following the analysis, the following four themes were developed: (1) Self-efficacy; (2) Knowledge; (3) Behavior; and (4) Acceptability.

#### 3.4.1. Theme 1: Self-Efficacy

Across the included studies, patient access to pancreatic cancer-related information was commonly reported. One of the strongest findings was the consistent demand from patients with pancreatic cancer for clear, comprehensive, and trustworthy information. Enzinger et al. [28] reported that more than 80% of participants wanted as much detail as possible about prognosis, life expectancy, treatment effects, and likely outcomes before beginning therapy. Similarly, the Animated Pancreas Patient (APP) digital resource evaluated by Munigala et al. [30] demonstrated both the scale of this demand and the potential reach of such digital interventions. The platform attracted more than 2.7 million unique users across 161 countries, over half of whom were patients themselves. Among those who provided feedback, 91% reported learning new information and 94% indicated an intention to use this knowledge to better manage their cancer or to initiate more informed discussions with their clinicians. Across studies, high levels of information-seeking and self-reported use of educational resources were observed among patients.

However, feeling informed did not always equate to being fully prepared for treatment and recovery. O’Connor et al. [31] explored preparedness among individuals undergoing pancreatic or hepatopancreatobiliary surgery and found that most felt moderately to well informed about postoperative pain and the use of analgesia, reflecting areas that were routinely emphasized by care teams. Yet, the same study revealed substantial gaps in understanding of longer-term aspects of recovery such as discharge planning, the need for enzyme replacement therapy, anticoagulation or vaccination following splenectomy, and the likely role of case managers. Some patients reported receiving inconsistent or contradictory information from different healthcare professionals, which undermined their sense of preparedness. The study reported lower levels of preparedness for longer-term aspects of recovery compared with perioperative pain management.

Several studies also linked information provision to behavioral intentions and proactive engagement. Enzinger et al. [28] found that patients who accessed enhanced educational resources were more likely to demonstrate accurate understanding of chemotherapy side effects compared to those receiving usual care, even if their expectations of benefit and overall distress levels were unchanged. Munigala et al. [30] similarly demonstrated that digital resources can support behavioral change, with most participants reporting an intention to act on what they had learned. Evidence from Anderson et al. [25], although conducted in a public awareness rather than patient sample, reinforces this point: participants who engaged with a serious game about pancreatic cancer symptoms not only showed statistically significant increases in knowledge but also a significant rise in their stated willingness to seek medical advice if they experienced relevant symptoms. Across these studies, educational interventions were associated with reported increases in understanding and stated intentions to seek medical advice.

Despite these benefits, education alone did not consistently alleviate emotional distress or anxiety. Enzinger et al. [28] found that additional educational resources did not reduce levels of distress compared to standard information, highlighting the limits of knowledge alone in addressing the emotional burden of cancer. Similarly, O’Connor et al. [31] reported that older patients often felt only somewhat prepared for surgery, which may reflect heightened vulnerability and anxiety in this group that information alone cannot fully mitigate. Educational interventions were not associated with reductions in distress or anxiety in the included studies

The role of digital platforms emerged as particularly important in expanding access to patient-centred education. Anderson et al. [25] demonstrated how interactive formats such as web-based serious games can effectively increase awareness and encourage behavioral change. Participants not only showed statistically significant improvement in symptom recognition and disease understanding but also reported increased motivation to seek medical advice if symptoms occurred. Similarly, Munigala et al. [30] showed that large-scale dissemination of multimedia educational resources, through the APP platform, can reach global audiences of patients, caregivers, and healthcare providers. The majority of users reported knowledge gains and intended to act on what they had learned, although viewer retention averaged only around 50% of total content length. Engagement metrics, knowledge gains, and retention data were reported for digital interventions, with variability across platforms [29].

Across studies, patients reported high information needs and self-reported increases in understanding following educational interventions.

#### 3.4.2. Theme 2: Knowledge

Knowledge and awareness outcomes were commonly reported across the included studies [25,26,28,30,32,33]. Findings consistently demonstrate that structured educational interventions substantially increase knowledge of pancreatic cancer symptoms, treatments, and management across diverse audiences. Although delivery methods ranged from traditional teaching (*n* = 2) to innovative digital formats such as games (*n* = 1), animations (*n* = 1), and virtual reality (*n* = 1), all interventions reported pre/post changes in knowledge-related outcomes. This impact was evident across both professional and public education settings, though the learning objectives and contexts differed.

Several studies targeting healthcare students and professionals demonstrated significant post-intervention improvements in knowledge and confidence. Bass et al. [26] used the Individual and Team Readiness Assurance Tests (iRAT/tRAT) to assess student learning during a gastrointestinal module, showing mean score improvements from 75% to over 98%. The use of structured multiple-choice questions (MCQs) before and after team discussion demonstrated the impact of collaborative learning on both factual recall and confidence. Würstle et al. [33] employed a 10-item pre/post knowledge questionnaire to evaluate understanding of pancreatic anatomy, pathology, and treatment with scores rising from 4.0 to 7.3 out of 10, alongside statistically significant rises in self-efficacy and motivation. Similarly, Phillips et al. [32] used a validated 20-item multiple-choice test to assess dietitian training, reporting performance improvements from 11% to 94% above the competence threshold following a structured programme, while Würstle et al. [33] reported that immersive VR enabled students to link theoretical learning concepts to clinical practice. Similarly, Phillips et al. [32] found that post-intervention testing showed a higher proportion of participants achieving the predefined competence threshold.

In contrast to professional education, patient- and public-focused interventions aimed to enhance awareness of symptoms, treatment options and self-management. Anderson et al. [25] implemented a pre/post symptom awareness questionnaire within a public facing web-based serious game, measuring recognition of correct and incorrect symptoms and reporting a large mean increase of 13.4 points (Cohen’s d = 1.43, as reported by the study authors). Munigala et al. [30] assessed user-reported learning and behavioral intention of patients and their caregivers via structured surveys embedded in the APP digital platform, while Enzinger et al. [28] conducted a randomized trial, involving patients, comparing standard information with multimedia educational booklets and videos, evaluating knowledge and understanding through structured questionnaires. While assessment tools were not standardized, all demonstrated measurable gains in disease understanding and confidence in self-management.

Beyond quantitative improvements, several studies suggested that learning translated into deeper conceptual understanding. Anderson et al. [25] found that participants became better able to differentiate relevant from irrelevant symptoms.

Overall, the measurement of knowledge and awareness showed considerable conceptual overlap across studies. Most assessed pre/post knowledge gains or perceived learning using bespoke questionnaires, with limited standardization of tools. Despite this variability, across studies, improvements in knowledge-related outcomes were reported. However, few studies examined long-term retention or behavioral outcomes, highlighting the need for validated instruments and longitudinal evaluation.

#### 3.4.3. Theme 3: Behavior

Several studies reported changes in self-reported behavioural intentions or practice-related outcomes. However, the extent to which knowledge translated into consistent behaviour varied, with some studies highlighting promising shifts and others revealing persistent gaps between evidence and practice.

Among public- and patient-focused studies, the link between education and behavioral intention was particularly clear. Anderson et al. [25] showed that participants who engaged with a serious game designed to increase pancreatic cancer awareness not only improved their symptom recognition but also reported a statistically significant rise in their willingness to seek medical help. Help-seeking intention scores increased by an average of 4.5 points, with a large effect size (Cohen’s d = 1.10, as reported by the study authors). Munigala et al. [30] provided further support for this link through their evaluation of the APP platform. Among participants who provided feedback, 94% indicated a commitment to act on the knowledge gained, whether through engaging more actively in discussions with their clinicians or using the information to manage their illness more effectively. The studies reported increases in stated help-seeking intentions and intended use of information.

Evidence from studies with patients also highlighted how education can shape expectations and preparedness for treatment. O’Connor et al. [31] found that patients who reported feeling well-informed were more confident about participating in aspects of their care, such as pain management and postoperative recovery. However, many also identified areas where a lack of education left them unprepared for discharge planning and long-term self-management. Enzinger et al. [28] similarly demonstrated that while patients valued detailed information about chemotherapy side effects and treatment outcomes, educational interventions did not significantly alter distress levels or perceptions of prognosis. Reported preparedness varied across domains of care, and distress levels were not significantly different between intervention and control groups.

While patient and public interventions demonstrated clear links between education and behavioral intention, the translation of learning into professional practice revealed a more complex picture. Barnes et al. [27] identified gaps between oncologists’ treatment choices and evidence-based guidelines, even when relevant data was available. For example, nearly half of the participants did not select an evidence-based adjuvant chemotherapy regimen following resection, and many recommended inappropriate second-line regimens for patients with neuropathy. In contrast, Phillips et al. [32] provided evidence that structured training for dietitians could rapidly align practice with standards. After the intervention, 94% of participants achieved competence above the pre-set threshold, with nearly one-fifth reaching specialist level. Variation in treatment selection was observed despite guideline availability, while post-intervention competence improved in the dietitian training study.

The potential for education to shape professional behaviour was also evident in studies with medical students. Bass et al. [26] demonstrated that students not only performed better in team-based learning compared to individual assessments but also rated the activity as highly effective for learning. Similarly, Würstle et al. [33] found that virtual reality simulations not only increased factual knowledge but also motivated students to pursue further learning about pancreatic cancer. Students reported increased motivation to engage with pancreatic cancer-related learning following the interventions.

However, the evidence also illustrates that educational interventions alone are not always sufficient to guarantee consistent changes in practice. Few studies assessed whether reported intentions translated into observable behavioural or clinical outcomes.

#### 3.4.4. Theme 4: Acceptability

A recurring strength across the included studies was the high level of acceptability and engagement with educational interventions. Whether delivered in traditional face-to-face formats, interactive team-based sessions, or through innovative digital media, most participants reported positive experiences and satisfaction with the resources provided. High levels of satisfaction and engagement were reported across intervention types.

Among healthcare students, interactive pedagogies were particularly well received. Bass et al. [26] found that over 90% of medical students agreed or strongly agreed that a team-based learning session on gastrointestinal topics, which included pancreatic cancer, was an effective method for learning. On a five-point Likert scale, the activity achieved a mean rating above 4.0, higher than any other instructional method evaluated at the institution at that time. Students not only performed better in teams compared to individual assessments but also expressed that the activity was both enjoyable and useful, highlighting how collaborative approaches can encourage active participation and deepen engagement. Würstle et al. [33] reported similarly positive responses to a virtual reality simulation, with nearly 88% of participants describing themselves as fully engaged and over 80% expressing interest in further VR-based teaching. These findings indicate that active, immersive methods are particularly valued by students, who may find traditional didactic teaching less stimulating.

Engagement and satisfaction were similarly strong among professional learners. Phillips et al. [32] reported that nearly 98% of dietitians rated all aspects of a national training course as ‘good’ or ‘excellent’. The few negative comments were isolated and largely related to course pacing rather than dissatisfaction with the content or delivery. The high satisfaction rate, combined with measurable improvements in competence, highlights the acceptability of targeted educational initiatives among professional groups and suggests strong potential for scalability. Importantly, the course not only delivered knowledge but was perceived as practical and relevant.

For patient and public audiences, engagement centred on usability, accessibility, and perceived relevance. Anderson et al. [25] found that participants rated the usability of a pancreatic cancer awareness game highly, with a mean System Usability Scale (SUS) score of nearly 60 out of 70, alongside positive star ratings, where most participants awarded four or five stars. Participants described the game as easy to use and valuable for learning, and their willingness to recommend it to others suggests that gamified approaches can be both engaging and credible tools for health education. Similarly, Munigala et al. [30] demonstrated that digital animations and videos were not only widely accessed—garnering millions of views globally—but also positively received by patients, caregivers, and healthcare providers. More than 90% of those who provided feedback reported learning something new, and most expressed intentions to act on the knowledge.

However, while engagement was generally high, some studies identified challenges and barriers. In the virtual reality intervention by Würstle et al. [33], around 15% of students reported nausea or ‘cybersickness’, and a small proportion described feeling stressed by the immersive environment. Although these experiences did not significantly affect overall learning outcomes, they highlight the need for careful attention to usability and participant comfort when designing technology-enhanced learning. Similarly, O’Connor et al. [31] reported that although patients felt well prepared for some aspects of surgery, inconsistent messages from healthcare providers led to frustration and reduced trust, illustrating that engagement can be undermined by lack of coherence in educational delivery. Engagement also appeared to vary by format and audience. Garikipati et al. [29], in their analysis of pancreatic cancer-related YouTube videos, found that patient-uploaded videos attracted significantly more likes and comments than those uploaded by healthcare professionals or societies, reflecting the strong resonance of personal narratives. Yet the average quality of educational content, as assessed against evidence-based criteria, was low, raising concerns about the potential for misinformation to attract higher engagement than accurate but less compelling professional resources.

## 4. Discussion

Interpretation of outcomes was informed by study quality, with greater confidence placed in findings from randomized and multi-site studies than from small, uncontrolled pre–post designs. Different to prior reviews focused on supportive care or shared decision-making [14], this review uniquely maps educational interventions across all populations targeting healthcare students, professionals, patients, carers, and the public. Although the evidence base is small and heterogeneous, the included studies reported improvements in knowledge-related outcomes and self-reported confidence, and reported changes in behavioural intentions or practice-related outcomes across diverse populations. However, the evidence base remains fragmented, largely confined to high-income settings, and limited in methodological rigour. Most studies employed small samples, relied on bespoke and unvalidated assessment tools, and lacked longitudinal evaluation, making it difficult to determine the sustainability of learning outcomes. Few applied educational or behavioral theory to intervention design, which constrains comparability, generalizability, and replication. Greater use of validated instruments and theory-driven frameworks would substantially improve comparability and rigour across future research. Given the predominance of short-term assessments and uncontrolled designs, these findings should be interpreted as associations reported within individual studies rather than evidence of sustained or causal effects.

This review identified four interrelated themes that collectively describe the educational landscape in pancreatic cancer: (1) Self-efficacy; (2) Knowledge; (3) Behavior; and (4) Acceptability. These themes summarise reported outcomes relating to information needs and preparedness, knowledge-related outcomes, behavioural intentions or practice-related outcomes, and acceptability. Across these domains, digital and interactive approaches were associated with high engagement metrics and positive acceptability ratings in the included studies, while traditional structured programmes reported pre/post improvements in knowledge or competence measures among professional groups. However, methodological variability and a lack of standardized outcome measures limit direct comparison across studies. A more standardized approach to educational evaluation would improve comparability across studies and support more consistent reporting of outcomes.

In the wider context, cancer education has been associated with improvements in awareness, knowledge, and professional skills across multiple cancer contexts [34]. Reviews of oncology curricula consistently highlight gains in knowledge and communication skills when structured education is provided to medical, nursing, and allied health students [35,36]. Similarly, patient and carer education interventions in cancer have reported improvements in treatment adherence, symptom self-management, and satisfaction with care [37,38]. The findings of this review suggest that similar outcomes may be observed in pancreatic cancer education, although the current pancreatic cancer evidence base is smaller and more heterogeneous.

One explanation for this deficit is the distinctive challenge pancreatic cancer poses: late presentation, rapid progression, and poor prognosis. These factors may affect the timing, delivery, and evaluation of educational interventions. Patients and families often have limited time to assimilate information, while professionals may have fewer opportunities for longitudinal learning compared with higher-incidence cancers. Broader literature on rare and low-survival cancers shows similar patterns of unmet informational needs, diagnostic delays, and professional uncertainty [39,40]. Education interventions in other cancer contexts have reported reductions in decisional conflict and improvements in shared decision-making and help-seeking intentions [41,42]. Translating such approaches into pancreatic cancer contexts may be relevant to pancreatic cancer education; however, evidence of downstream clinical or system-level impact (e.g., earlier diagnosis or improved outcomes) was not assessed in the included pancreatic cancer studies [43].

A persistent cross-cutting theme within this evidence base is the gap between knowledge-related outcomes or stated intentions and measured behavioural outcomes. Although several studies reported improvements in engagement metrics, self-reported intentions, and selected practice-related outcomes, only a few evaluated whether these changes were sustained over time. This pattern is consistent with behavioral science frameworks such as COM-B and the Theory of Planned Behavior [44,45], which emphasize that knowledge and capability alone are insufficient without adequate environmental opportunity, reinforcement, and motivation. These frameworks highlight the importance of addressing contextual and system-level factors alongside education interventions to support evaluation of behavioural outcomes over time.

Broader oncology education has reported that interactive, problem-based approaches are associated with improvements in learning outcomes and may support practice change [46], and interventions linked to audit, feedback, or policy mandates have been associated with practice change [47]. For pancreatic cancer, embedding education within multidisciplinary training, continuing professional development (CPD), and survivorship care planning may support implementation and evaluation in practice settings. System-level reinforcement through clinical guidelines, performance monitoring, and leadership support may be relevant when evaluating behavioural outcomes beyond the immediate post-intervention period.

Digital modalities featured prominently across included studies, reflecting wider trends in both medical education and patient engagement. E-learning, simulation, gamification, and multimedia resources are now well established in oncology education, with systematic reviews reporting outcomes that are comparable to, and in some cases better than, face-to-face teaching [48,49]. For patients, online information and peer communities are increasingly central sources of information and support, though concerns about misinformation persist [50]. The variable quality of online pancreatic cancer content identified in this review echoes broader findings on across digital platforms where patient generated narratives often attract more engagement than professionally curated materials [51]. This is associated with both opportunities and risks; while relatable, emotionally resonant stories enhance engagement, they may also disseminate inaccurate or incomplete information. Quality assurance, co-design with patients, and integration with trusted health systems are relevant design considerations. Also noteworthy, the virtual reality intervention reported a 15% incidence of nausea or cybersickness, highlighting the need for future VR-based education to incorporate mitigation strategies such as shorter exposure durations, optional non-immersive alternatives, and user-controlled navigation to improve accessibility. Moreover, digital approaches must account for disparities in digital literacy and access, particularly among older adults and socioeconomically disadvantaged populations, to prevent widening educational inequities [52,53]

These findings are relevant to policy and practice discussions about pancreatic cancer education. As a scoping review, this study aimed to map and synthesise existing educational interventions in pancreatic cancer rather than to evaluate implementation pathways, funding mechanisms, or workforce planning. Questions relating to who should lead, fund, or deliver educational initiatives, at national or global levels, were beyond the scope of the included studies and were not addressed in the available evidence. The findings should therefore be interpreted as an overview of what has been evaluated to date, rather than as an implementation framework. That stated, cancer education is explicitly recognised in national and international frameworks as a mechanism associated with improvements in health literacy, engagement, and aspects of care in other cancer contexts. The NHS Long Term Plan prioritizes early diagnosis and patient information, while the EU Beating Cancer Plan emphasizes health literacy and digital innovation [54]. The World Health Organization’s (WHO) global strategy for cancer control identifies education as a core pillar for reducing avoidable mortality [55]. Yet pancreatic cancer remains under-represented within these initiatives, reflecting both its lower incidence and entrenched fatalism. Given its poor prognosis and rapid disease trajectory, embedding pancreatic cancer education across professional curricula, public awareness campaigns, and carer support programmes may be considered in future educational and policy initiatives.

### 4.1. Recommendations

Based on gaps identified in the included studies, future work may consider: (1) development of inter-disciplinary programmes that reach professionals, patients, and carers simultaneously; (2) integration of pancreatic cancer modules into undergraduate and postgraduate curricula; (3) development and evaluation of accessible, engaging, and quality-assured digital platforms; and (4) rigorous evaluation, including longitudinal research, through mixed-methods and, where feasible, randomized designs with long-term follow-up. To support comparability and interpretability across studies, future interventions should be explicitly grounded in educational or behavioral theory, clearly specifying learning objectives, mechanisms of change, and intended outcomes. Importantly, most current evidence originates from high-income countries. Further research in low- and middle-income settings is needed, as awareness and diagnostic challenges are often greater and infrastructure for specialist care more limited [5]. Global collaboration, knowledge-sharing, and capacity-building may support broader development and evaluation. This may include partnerships between academic institutions, cancer societies, and digital health organizations to share curricula, resources, and evaluation tools across contexts.

Overall, this review highlights both promise and need. Pancreatic cancer education can demonstrably improve knowledge and confidence across diverse audiences, and digital platforms offer scalable and cost-effective reach. Yet sustained effects on professional practice and patient outcomes remain unproven. Moving forward, embedding education within systemic cancer control policies, expanding research beyond high-income contexts, and rigorously evaluating interventions will be essential. In a disease characterized by late presentation and poor survival, education remains one of the most accessible strategies to promote earlier help-seeking, strengthen professional preparedness, and support families in care. The challenge is to build on early successes and establish pancreatic cancer education as an integral component of global cancer policy and practice.

### 4.2. Strengths and Limitations

This review has several strengths. To our knowledge, it is the first to systematically map and synthesize peer-reviewed evidence on pancreatic cancer education across healthcare students, professionals, patients, carers and the public. The review followed a prospectively registered protocol and adhered to established methodological guidance, including the JBI framework and PRISMA-ScR checklist, ensuring transparency and reproducibility. The comprehensive search across four major databases, dual independent screening, and use of structured data-charting further enhance the robustness of the review. By including diverse study designs, the review captured the breadth of educational interventions in this emerging field, spanning traditional teaching, digital innovations, and patient-centred resources. Together, these methodological strengths increase confidence in the completeness and credibility of the evidence map produced.

However, several limitations should be acknowledged. Most included studies evaluated immediate or short-term outcomes; therefore, this review cannot determine whether educational interventions lead to sustained behaviour change, earlier diagnosis, or improved clinical outcomes. Further, only nine studies met eligibility criteria, reflecting the paucity of research in this area. Most originated from high-income countries, limiting generalizability to low- and middle-income settings where awareness and resource needs may be greatest. The evidence base was heterogeneous in population, intervention, and outcomes, precluding meta-analysis and limiting conclusions about effectiveness. Many studies relied on short-term, self-reported outcomes, with few incorporating rigorous experimental designs or long-term follow-up. Statistical outcomes are reported as presented in the original studies; however, heterogeneity in outcome measures and reporting methods limits direct comparison across interventions. Future research would benefit from greater use of standardised, validated outcome measures to improve comparability across studies. A core outcome set for pancreatic cancer education could include symptom recognition accuracy; knowledge retention at predefined time points; self-efficacy using validated scales; behavioural intention measures; and, where feasible, downstream outcomes such as referral patterns or time to diagnosis. Adoption of consistent metrics would strengthen synthesis and support progression toward effectiveness evaluation

In addition, grey literature was excluded, which may have omitted potentially relevant practice-based or innovative educational resources. The absence of standardized, validated outcome measures further limits comparability and reduces the ability to assess the true magnitude of educational effects. The review does not address feasibility, cost, workforce capacity, or responsibility for implementation of educational interventions. Many recommendations commonly proposed in pancreatic cancer education, including multidisciplinary training, digital platforms, and global collaboration, are well recognised but remain challenging to operationalise, particularly in low- and middle-income settings. The absence of implementation and economic evaluations within the included studies limits conclusions regarding scalability or sustainability. Collectively, these limitations highlight the need for more robust, globally inclusive research to strengthen the evidence base and guide future educational development in pancreatic cancer.

## 5. Conclusions

This scoping review demonstrates the emerging potential of educational interventions in pancreatic cancer, showing consistent improvements in knowledge, confidence, and engagement across diverse audiences. Digital platforms in particular offer scalable opportunities for dissemination and reach, although challenges regarding content quality, accessibility, and sustained engagement persist. Despite a limited and fragmented evidence base, these findings highlight education’s vital role in improving outcomes through earlier recognition, shared decision-making, and adherence to guideline-based care. Importantly, education alone cannot be a substitute for psychosocial support, counselling, or holistic care, which remain essential to addressing the broader emotional and practical needs of patients and families. High-quality educational strategies must therefore be embedded within multidisciplinary models of care to maximize their impact.

Future research should prioritize rigorous, interdisciplinary, and globally relevant approaches to ensure that pancreatic cancer education is embedded as a core component of comprehensive cancer control strategies worldwide. Long-term evaluation of educational impact, real-world behavioral change, and downstream effects on diagnostic timeliness will be crucial to advancing this field.

## Figures and Tables

**Table 1:** Eligibility criteria structured using the PCC framework as recommended by the JBI [16].

Element	Description	Application in this Review
Population	The group(s) targeted by the intervention or focus of the evidence.	- Healthcare students (undergraduate or postgraduate in medicine, nursing, or pharmacy) - Qualified healthcare professionals (e.g., nurses, doctors, pharmacists, radiotherapists) - Patients with pancreatic cancer - Carers/family members - Members of the general public
Concept	The main idea or phenomenon of interest.	- Pancreatic cancer education, defined as interventions or resources designed to improve awareness, knowledge, attitudes, or self-efficacy relating to pancreatic cancer - Studies included if pancreatic cancer-specific outcomes were reported, even within broader cancer education
Context	The setting, environment, or circumstances in which the concept is explored.	- Any healthcare or educational setting (e.g., university, hospital, community, or home) - Any delivery mode (e.g., face-to-face teaching, online modules, written materials, simulation, scenario-based or gamified learning)

**Table 2:** EMBASE Search String.

EMBASE	
S1 Pancreatic cancer	95,947
S2 Pancreatic neoplasms	1694
S3 pancreatic tumours	1035
S4 pancreatic adenocarcinoma	18,228
S5 cancer of the pancreas	2805
S6: S1 OR S2 OR S3 OR S4 OR S5	108,007
S7 Healthcare students	1675
S8 Healthcare professional students	139
S9 Pre-registration healthcare students	14
S10 Nursing students	23,328
S11 Medical students	71,054
S12 Pharmacy students	7112
S13 Healthcare professionals	68,537
S14 Registered healthcare professionals	33
S15 Nurse	384,553
S16 Registered nurse	10,553
S17 Doctor	214,124
S18 Medical doctor	2701
S19 Physician	659,925
S20 Pharmacist	120,495
S21 Registered pharmacist	68
S22 Public	1,136,669
S23 General public	20,963
S24 Patients	11,682,553
S25: S7 OR S8 OR S9 OR S10 OR S12 OR S13 OR S14 OR S15 OR S16 OR S17 OR S18 OR S19 OR S20 OR S21 OR S22 OR S23 OR S24 OR S25 OR S26	13,272,559
S26 Education	1,533,016
S27 Intervention	1,465,931
S28 Teaching	311,080
S29 Learning	896,733
S30: S26 OR S27 OR S28 OR S29	3,682,749
S31: S6 AND S25 AND S30	4641

**Table 3:** Quality appraisal outcomes using JBI Critical Appraisal Checklists and the Mixed Methods Appraisal Tool (MMAT) [20,24].

Study	Design/Appraisal Tool	Key Strengths	Main Limitations	Overall Appraisal Outcome
Anderson et al. [25]	Quasi-experimental (JBI checklist)	Clear aims; large sample; appropriate statistics	No control group; potential self-selection bias	Moderate–High
Bass et al. [26]	Educational intervention (pre–post, JBI checklist)	Real-world educational setting; good alignment with learning objectives	Single cohort; limited generalizability	Moderate
Barnes et al. [27]	Cross-sectional survey (JBI checklist)	National sample; relevant to practice	Self-reported data; potential recall bias	Moderate
Enzinger et al. [28]	Randomized controlled trial (JBI RCT checklist)	Strong design; clear randomization; robust analysis	Limited follow-up; self-reported outcomes	High
Garikipati et al. [29]	Content analysis (qualitative JBI checklist)	Systematic coding; transparency of process	No inter-rater reliability reported	Moderate
Munigala et al. [30]	Retrospective analytics (observational JBI checklist)	Very large dataset; real-world evidence	Limited demographic data; potential confounding	Moderate–High
O’Connor et al. [31]	Cross-sectional survey (JBI checklist)	Clear aim; relevant to patient preparedness	Small sample; no inferential analysis	Moderate–Low
Phillips et al. [32]	Pre–post evaluation (JBI quasi-experimental checklist)	Large multi-site sample; validated measures	No control group; response rate variation	Moderate–High
Würstle et al. [33]	Prospective single arm (JBI quasi-experimental checklist)	Objective pre/post testing; high engagement	No comparator; possible novelty bias	Moderate–High

## Data Availability

Data sharing is not applicable to this article as no new data were created or analyzed in this study. The review protocol is publicly available on the Open Science Framework (OSF) registry.

## Supplementary Materials

The following supporting information can be downloaded at: https://www.mdpi.com/article/10.3390/curroncol33010033/s1, Table S1: Preferred Reporting Items for Systematic reviews and Meta-Analyses extension for Scoping Reviews (PRISMA-ScR) Checklist. Table S2: Data Extraction Table.

## Abbreviations

The following abbreviations are used in this manuscript:

UK	United Kingdom
GP	General Practitioner
JBI	Joanna Briggs Institute
PCC	Population–Concept–Context
PRISMA-ScR	Preferred Reporting Items for Systematic reviews and Meta-Analyses extension for Scoping Reviews
OSF	Open Science Framework
RCT	Randomized Control Trial
MMAT	Mixed Methods Appraisal Tool
TBL	Team-based learning
HPB	Hepatopancreatobiliary
APP	Animated Pancreas Patient
iRAT/tRAT	Individual/team readiness assurance tests
VR	Virtual reality
WHO	World Health Organization
