# Supplementary material for: Pancreatic Cancer Education: A Scoping Review of Evidence Across Patients, Professionals and the Public
Source: Curr Oncol. 2026 Jan 8;33(1):33. doi: 10.3390/curroncol33010033 (PMC12840498; doi:10.3390/curroncol33010033)
Supplement: Supplementary file 1 [file curroncol-33-00033-s001.zip › curroncol-4039689-supplementary.pdf]

Table S1: Preferred Reporting Items for Systematic reviews and Meta-Analyses extension for
Scoping Reviews (PRISMA-ScR) Checklist

SECTION	ITEM	PRISMA-ScR CHECKLIST ITEM	REPORTED ON PAGE #
**TITLE**			
Title	1	Identify the report as a scoping review.	1
**ABSTRACT**			
Structured summary	2	Provide a structured summary that includes (as applicable): background, objectives, eligibility criteria, sources of evidence, charting methods, results, and conclusions that relate to the review questions and objectives.	1
**INTRODUCTION**			
Rationale	3	Describe the rationale for the review in the context of what is already known. Explain why the review questions/objectives lend themselves to a scoping review approach.	2
Objectives	4	Provide an explicit statement of the questions and objectives being addressed with reference to their key elements (e.g., population or participants, concepts, and context) or other relevant key elements used to conceptualize the review questions and/or objectives.	3
**METHODS**			
Protocol and registration	5	Indicate whether a review protocol exists; state if and where it can be accessed (e.g., a Web address); and if available, provide registration information, including the registration number.	4
Eligibility criteria	6	Specify characteristics of the sources of evidence used as eligibility criteria (e.g., years considered, language, and publication status), and provide a rationale.	4
Information sources*	7	Describe all information sources in the search (e.g., databases with dates of coverage and contact with authors to identify additional sources), as well as the date the most recent search was executed.	5
Search	8	Present the full electronic search strategy for at least 1 database, including any limits used, such that it could be repeated.	Supplementary files page 3
Selection of sources of evidence†	9	State the process for selecting sources of evidence (i.e., screening and eligibility) included in the scoping review.	4
Data charting process‡	10	Describe the methods of charting data from the included sources of evidence (e.g., calibrated forms or forms that have been tested by the team before their use, and whether data charting was done independently or in duplicate) and any processes for obtaining and confirming data from investigators.	5
Data items	11	List and define all variables for which data were sought and any assumptions and simplifications made.	Supplemtary files pages 5-11
Critical appraisal of individual sources of evidence§	12	If done, provide a rationale for conducting a critical appraisal of included sources of evidence; describe the methods used and how this information was used in any data synthesis (if appropriate).	7-8

Synthesis of results	13	Describe the methods of handling and summarizing the data that were charted.	5

SECTION	ITEM	PRISMA-ScR CHECKLIST ITEM	REPORTED ON PAGE #
**RESULTS**			
Selection of sources of evidence	14	Give numbers of sources of evidence screened, assessed for eligibility, and included in the review, with reasons for exclusions at each stage, ideally using a flow diagram.	6-7
Characteristics of sources of evidence	15	For each source of evidence, present characteristics for which data were charted and provide the citations.	8-9
Critical appraisal within sources of evidence	16	If done, present data on critical appraisal of included sources of evidence (see item 12).	7-8
Results of individual sources of evidence	17	For each included source of evidence, present the relevant data that were charted that relate to the review questions and objectives.	9-14
Synthesis of results	18	Summarize and/or present the charting results as they relate to the review questions and objectives.	6
**DISCUSSION**			
Summary of evidence	19	Summarize the main results (including an overview of concepts, themes, and types of evidence available), link to the review questions and objectives, and consider the relevance to key groups.	15
Limitations	20	Discuss the limitations of the scoping review process.	16-17
Conclusions	21	Provide a general interpretation of the results with respect to the review questions and objectives, as well as potential implications and/or next steps.	17
**FUNDING**			
Funding	22	Describe sources of funding for the included sources of evidence, as well as sources of funding for the scoping review. Describe the role of the funders of the scoping review.	18

JBI = Joanna Briggs Institute; PRISMA-ScR = Preferred Reporting Items for Systematic reviews and Meta-Analyses extension for Scoping Reviews.

* Where *sources of evidence* (see second footnote) are compiled from, such as bibliographic databases, social media platforms, and Web sites.

† A more inclusive/heterogeneous term used to account for the different types of evidence or data sources (e.g., quantitative and/or qualitative research, expert opinion, and policy documents) that may be eligible in a scoping review as opposed to only studies. This is not to be confused with *information sources* (see first footnote).

‡ The frameworks by Arksey and O'Malley (6) and Levac and colleagues (7) and the JBI guidance (4, 5) refer to the process of data extraction in a scoping review as data charting.

§ The process of systematically examining research evidence to assess its validity, results, and relevance before using it to inform a decision. This term is used for items 12 and 19 instead of "risk of bias" (which is more applicable to systematic reviews of interventions) to include and acknowledge the various sources of evidence that may be used in a scoping review (e.g., quantitative and/or qualitative research, expert opinion, and policy document).

From: Tricco AC, Lillie E, Zarin W, O'Brien KK, Colquhoun H, Levac D, et al. PRISMA Extension for Scoping Reviews (PRISMAScR): Checklist and Explanation. Ann Intern Med. 2018;169:467–473. doi: 10.7326/M18-0850.

Table S2: Data Extraction Table

Citation	Aim(s)	Country of origin	Population	Concept	Context / setting	Study design	Data collection	Data analysis	Key findings	Limitations / notes
Anderson et al. (2024)<br><br>'Co-design and evaluation of a digital serious game to promote public awareness about pancreatic cancer'<br><br>BMC Public Health, 24, 570.	To co-design and evaluate whether a short, browser based 'serious game' improves public awareness of pancreatic cancer symptoms and intentions to seek help.	UK (NI) with international recruitment via web.	N=727 public participants; paired pre/post data available for N=489 (96% from Northern Ireland; 92% female; majority aged 18–25; 78% student health professionals).	Public symptom awareness, self-efficacy and help-seeking intentions related to pancreatic cancer; usability/acceptability of a co-designed digital game.	Open-access HTML5 web app during World Pancreatic Cancer Awareness Month (Nov 2022); co-designed with people with lived experience, advocates, and HCPs.	Quasi-experimental pre-/post-test without control; usability survey (System Usability Scale, adapted).	Embedded pre/post test questionnaires: 20 symptom awareness items, 7 help-seeking/self-efficacy items; demographics; post-game System Usability Scale; and star rating.	Paired t-tests for pre/post change; Cohen's d; independent t-tests across subgroups (age, gender, prior experience).	Symptom awareness increased significantly: mean 47.08→59.10.<br><br>Help-seeking intentions increased: mean 12.83→17.03.<br><br>No significant differences in improvements by prior experience, gender, or age. Item-level awareness improvements for true symptoms (e.g., jaundice, pale/smelly stools, indigestion, diabetes, upper abdominal/middle back pain, weight loss, low mood).<br><br>Usability: 67.6% gave 4–5 stars (≈22% 4★, 46% 5★).<br><br>Participants reported the game was easy to use, educational, and recommended to others. Co-design ensured content relevance; delivery via web broadened access.	Convenience sample caused over-representation of young female/student healthcare professional which limits generalisability.<br><br>No control group.<br><br>Immediate post-test only (no retention) and 27.6% missing post-tests.
Barnes et al. (2019)<br><br>'Identification of Educational Gaps Among Oncologists Who Manage Patients with Pancreatic Cancer'<br><br>Journal of Gastrointestinal Cancer, 50, pp. 84–90.	To assess US oncologists' pancreatic ductal adenocarcinoma (PDA) practice patterns across disease stages and identify gaps vs evidence-based guidelines to inform continuing medical education (CME) priorities.	USA	N=150 US medical oncologists (19% academic, 81% community)<br><br>Median 80 pts/week; 10 PDA pts/month; 64% of PDA pts receiving 2nd-line therapy.	Assessing guideline adherence in adjuvant therapy selection, 2nd-line metastatic choices post, and diagnostic work-ups for suspected metastatic PDA.	Online case-vignette survey (June–July 2016); $50 incentive; vignettes spanned resectable, locally advanced cancers to metastatic progression.	Cross-sectional survey using validated case vignettes (3 separate scenarios with MCQs).	Self-administered responses to MCQs on regimens, tests, referrals; demographics; practice setting and PDA patient volume.	Descriptive statistics (frequencies/means) in SPSS; comparison to then current ASCO/NCCN guidance.	Case 1 (adjuvant chemo after distal pancreatectomy): 44% chose non-evidence-based regimens (e.g., FOLFIRINOX, nab-paclitaxel/gemcitabine) not yet established in adjuvant setting at that time; only 56% selected evidence-based options (gemcitabine, 5-FU, or gemcitabine+capecitabine per ESPAC-4).<br><br>Case 2 (chemo post pancreatectomy with neuropathy, progression to metastatic): only 23% chose evidence based options; 66% chose other 5-FU based regimens, and 35% overall chose oxaliplatin-containing	Survey self-report (intent, not observed behaviour).<br><br>Knowledge may have evolved quickly.<br><br>No inferential testing.<br><br>Case simplifications.<br><br>Potential response bias despite random sampling.

									regimens despite neuropathy risk.<br><br>Case 3 (suspected metastatic PDA): 88% recommended biopsy (67% liver), but only 53% ordered chest imaging; 35% ordered LFTs; only 34% ordered all three (biopsy + chest imaging + LFTs).<br><br>Clinical trial referral was ≤5% across scenarios despite guideline encouragement.	
Bass et al. (2018)<br><br>'A Case of Pancreatic Cancer: Abdominal Anatomy Team-Based Learning (TBL) Module for Medical Students'<br><br>MedEdPORTAL, 14, 10700.	To integrate gross anatomy education with radiology and clinical decision making training using a pancreatic cancer case-based TBL and evaluate student learning and perceptions.	USA	First-year medical students (N= 186 per cohort); sessions run with one-third of cohort per sitting (10–11 teams, 5–6 students/team).	Applied abdominal anatomy (retroperitoneum, posterior abdominal wall, neurovasculature) via an unfolding pancreatic cancer case study including imaging, surgery, palliation.	Preclinical GI module; co-facilitated by anatomist, radiologist and surgeon; audience response; and application MCQs with simultaneous reporting.	Educational intervention report with routine program evaluation (no control); repeated annual use since 2014.	Individual readiness assurance test (iRAT) and team readiness assurance test (tRAT) scores (10 items), application question performance, end-of-course Likert evaluations of effectiveness; and facilitator observations.	Descriptive stats for iRAT/tRAT; comparison of individual vs team performance; proportion in agreement that the activity was effective.	Teams outperformed individuals: mean iRAT =75% vs tRAT = 99% (2016–2017), showing collaborative gains.<br><br>Over 90% of students agreed/strongly agreed the TBL was effectual for learning (M=4.1–4.2/5).<br><br>Most challenging application item related to palliative celiac plexus block; imaging identification fostered anatomy-radiology integration. The format promoted vertical integration of basic and clinical sciences early in training.	No pre/post knowledge test or control.<br><br>Limited generalisability.<br><br>High faculty time investment (≈12 hours across facilitators).<br><br>Edits to some RAT items post intervention limit item-level comparability.
Enzinger et al. (2020)<br><br>'Effectiveness of a Multimedia Educational Intervention to Improve Understanding of the Risks and Benefits of Palliative Chemotherapy in Patients with Advanced Cancer: A Randomized Clinical Trial'	To test whether regimen-specific booklets and videos featuring authentic patient narratives improve patients' understanding of palliative chemotherapy (PC) benefits/risks and decision quality.	USA	N=186 adults with metastatic colorectal (63%) or locally advanced/metastatic pancreatic cancer (37%) considering 1st or 2nd line PC.	Patient comprehension of curability and adverse effects; decision conflict/regret; communication satisfaction; emotional distress.	Pragmatic trial: intervention offered without mandated viewing; usual care = site materials; assessments at baseline, 2–4 weeks (post-decision), and 8–12	Multicentre; unblinded RCT; multiple imputation for missing data; powered to detect 50% relative increase in accurate expectations (assumed 40% baseline).	Surveys: **Cancer Care Outcomes Research and Surveillance Consortium (**CanCORS) item on cure likelihood (primary); treatment goals; regimen-specific adverse-effect expectations; Control Preferences;	Fisher's exact test; Chi-square test; Wilcoxon test; analysis of variance (ANOVA); multiple imputation (10 datasets) integrated via Rubin's rules.	Primary outcome (accurate expectation that chemotherapy is 'not at all likely' to cure): no difference (intervention 52.6% vs control 55.5%).<br><br>Accurate understanding of adverse effects trended higher with intervention (56.0% vs 40.2%).<br><br>No differences in decision conflict, decisional regret,	Low video uptake.<br><br>Highly educated academic cohort.<br><br>No enforcement of exposure.<br><br>Inability to disentangle 'hope' vs misunderstanding.

JAMA Network Open, 3(6), e208250.					weeks (follow-up).		PACE communication; Decisional Regret; functional assessment of cancer therapy-general (FACT-G).		communication satisfaction, or distress.<br><br>Uptake: 78% read booklet, 41% watched video; satisfaction ~7.8/10; explicit statements of non-curative intent did not increase distress. Interpretation: expectations for cure may reflect hope rather than knowledge deficits; integration with clinician/nurse teaching or delayed timing may be needed.	Potential contamination of usual care.<br><br>Unblinded design.
Garikipati et al. (2023)<br><br>'Assessing the Educational Value of Pancreatic Cancer Videos on YouTube'<br><br>Journal of Cancer Education.	To evaluate the quality, reliability, and educational usefulness of YouTube videos on pancreatic cancer accessible to the public.	USA	Convenience sample of top YouTube search results on 'pancreatic cancer'.	User-rated video content quality (accuracy, completeness, bias), potential for misinformation; alignment with evidence-based guidance.	Public platform (YouTube); cross sectional snapshot of available videos at time of search.	Content analysis using validated scoring tools (e.g., DISCERN, JAMA benchmarks, Global Quality Score [GQS]); categorisation by source and topic (symptoms, diagnosis, treatment, prognosis).	Video metadata (duration, views/likes), source type, content topics; independent reviewer ratings on quality; inter-rater agreement calculated.	Descriptive statistics; comparison of scores across source categories (academic/ society vs commercial/individual); correlation of popularity metrics with quality; IRR statistics.	A minority of videos met high standards for reliability/quality.<br><br>Academic/society uploads achieved higher DISCERN/JAMA/GQS than individual/commercial sources.<br><br>Popularity (views/likes) did not correlate with quality; several high-view videos contained omissions or inaccuracies (e.g., overstatement of cure/control).<br>Critical gaps in video content: limited discussion of PEI/PERT, clinical trials, prognosis communication, and supportive care; few videos referenced sources or dates.	Dynamic platform so content changes over time.<br><br>English-language bias.<br><br>Potential rater subjectivity despite validated tools.<br><br>Search algorithm personalization may affect reproducibility.
Munigala et al. (2022)<br><br>'Helping Patients Understand Pancreatic Cancer Using Animated Pancreas Patient	To assess whether an online animated educational module 'Animated Pancreas Patient'	USA	Patients with pancreatic cancer and/or caregivers who accessed APP	Digital patient education using animation/visual learning; impact on knowledge, satisfaction,	Open-access web modules/videos; asynchronous learning in	Retrospective observational analysis of usage metrics and post-exposure surveys;	Web analytics (views, time-on-page), user surveys on perceived knowledge gain, decision	Descriptive summaries; subgroup comparisons where applicable; no randomization.	Users reported improved understanding of diagnosis/treatment pathways and felt better prepared for discussions with clinicians.	Retrospective.<br><br>Self-selected users.<br><br>No objective knowledge

Education With Visual Formats of Learning'<br><br>Pancreas, 51(10), pp. 882–889.	(APP) improves patient understanding/satisfaction and supports decision making in pancreatic cancer.		website/YouTube; retrospective analytics and survey subset.	and decision support.	routine clinic pathways.	comparative impressions vs written materials.	confidence, satisfaction; optional open comments.		Modules facilitated family education.<br><br>Visual formats (animation, narration) perceived as clearer than text-only materials.<br><br>Reported higher satisfaction and usefulness for decision-making.<br><br>Clinics could integrate APP into pre-visit education and reinforce key messages.	testing or control.<br><br>Potential positivity bias.<br><br>Cannot infer causality.<br><br>Limited demographic capture.
O'Connor et al. (2020)<br><br>'Preparedness for Surgery: Analyzing a Quality Improvement Project in a Population of Patients Undergoing Hepato-Pancreatico-Biliary Surgery'<br><br>Clinical Journal of Oncology Nursing, 24(5), E65–E69.	To evaluate perceived preoperative preparedness and identify educational gaps among HPB surgery patients to inform improvements in pre-op teaching and discharge planning.	USA	N=50 postoperative HPB patients (pancreatic cancer 56%, ampullary/duodenal 14%, benign/premalignant 30%); mix of procedures (PPPD, distal pancreatectomy, classic Whipple, total pancreatectomy).	Patient-reported preparedness across domains: pain expectations/management, NPO/diet progression, ambulation, case management, discharge meds (enzymes, acid blockers), rehab referrals, follow-up calls.	Single high-volume HPB unit; existing materials included Lustgarten 'Navigating Pancreatic Cancer' book, pre-admission testing review; survey on POD 4–5.	Cross-sectional QI survey (8-item Likert 1–4) with open comments; chart abstraction for clinical characteristics.	Post-op bedside questionnaire; HER extraction of surgery type, LOS (mean 5.5 days), comorbidities; qualitative comments captured and recorded by nurses.	Descriptive statistics (mean per item); thematic collation of comments; no inferential testing.	Best-prepared domains: expectation of post-op pain (all 'well informed') and participation in own pain management; awareness of early ambulation and initial diet restriction was moderate-to-good.<br><br>Least-prepared domains: discharge planning logistics, role of case manager, discharge medications (PERT, acid suppression), and potential need for post-acute rehab—the lowest scoring item.<br><br>Qualitative themes: inconsistent messages from different providers; lack of pre-op information on drains/lines, ICU stay possibility, NPO mechanics; surprise regarding insulin/anticoagulation/TPN; vaccination needs after splenectomy.	Small convenience sample from single centre.<br><br>Instrument not validated beyond face/content.<br><br>Only post-op recall assessed.<br><br>Did not capture preferred learning formats.<br><br>QI (no IRB consent needed).
Phillips et al. (2022)<br><br>'Training 1,200 dietitians: An evaluation	To develop and evaluate an accessible training course	UK and Ireland	N=1,215 attendees across 32 venues	Foundational knowledge of pancreatic physiology, PEI	2.5-hour sessions (late afternoon/evening/ weekend)	Pre/post test educational evaluation; service	10-item MCQ questionnaire (max 43 marks) before/after;	Paired t-test; proportion achieving thresholds;	Knowledge improved substantially: mean 22.2±5.3 → 35.8±4.0.	No long-term retention or behaviour change assessment.

of a training course for non-specialist dietitians on the management of pancreatic exocrine insufficiency'<br><br>Clinical Nutrition Open Science, 44, pp. 155–162.	for non-specialist dietitians on PEI recognition and PERT use; to measure knowledge gains and course acceptability.		(2014–2018); n=998 completed paired pre/post questionnaires; mix of non-specialist dietitians and final-year undergraduates.	aetiology /signs, malabsorption, PERT dosing/ titration, basics of Type 3c diabetes, surgery, enteral feeding; case-based application.	to maximise access; unconditional educational grant (Mylan); delivered by specialist pancreatic dietitians; delegate packs provided.	evaluation (no formal ethics).	pre-set thresholds: 33% minimal baseline, 70% 'safe' non-specialist target, >93% 'specialist-level'; course evaluations (9 aspects; good/excellent/average/poor).	descriptive analysis of 7,430 evaluation points; qualitative comments summarised.	Proportion ≥70% rose from 11% to 94%; ≥93% rose from 0% to 20%.<br><br>97.8% evaluation ratings were 'good/excellent'; only 4 isolated 'poor' ratings (pace too fast).<br><br>Evening/weekend delivery enabled whole-team attendance.<br><br>Participants highlighted immediate practice changes and unmet undergraduate training needs ('didn't know what I didn't know').	Tool not externally validated.<br><br>Some sessions had lower completion due to logistics.<br><br>Potential sponsor-perception bias (unrestricted grant; no content input).
Würstle et al. (2024)<br><br>'Evaluation of a Virtual Reality–Based Open Educational Resource Software'<br><br>Journal of Medical Education and Curricular Development, 11, 23821205241234567.	To evaluate the effectiveness, usability, and factors influencing learning of a VR teaching course on pancreatic cancer built with the open-source EVENT tool.	Germany	N=117 medical students (after exclusions) from 6th–8th semesters; 85% no prior VR experience; ~84% no videogame experience.	VR-based, self-paced learning covering etiology, epidemiology, symptoms, diagnosis, histopathology, therapy, prognosis; embedded quizzes; self-efficacy; workload; usability.	Single-arm prospective course (90 min total; ~40 min VR) using Meta Quest headsets; on-site technical support; ~4 m² movement space per learner.	Pre/post knowledge test (10 MCQs); self-efficacy ratings; SUS usability; NASA-TLX workload; exploratory analysis of factors (VR experience, spatial awareness, motivation, cyber-sickness).	Pre/post tests; Likert questionnaires; SUS (10 items); NASA-TLX (7 items); free-text feedback; demographics.	Wilcoxon paired tests; Fisher/Chi square tests for categorical; Spearman correlations; two-sided α=0.05; normalized learning growth computed.	Knowledge improved from 4.0±1.2 to 7.3±1.1/10); normalized learning growth 55.9%.<br><br>Self-efficacy increased significantly (p<0.001).<br><br>Usability high: mean SUS 86.1 ('excellent'); SUS>68 associated with higher post-scores.<br><br>VR experience, age, gender, spatial imagination, motivation, and cyber-sickness did not materially affect outcomes.15.4% reported mild nausea without performance impact.<br><br>Stress from the teaching unit (not VR per se) correlated with slightly lower post-scores (7.0 vs 7.5; p=0.032).<br><br>85.5% felt they performed well; 81.2% wanted more VR	Single-arm (no control).<br><br>Single institution. Questionnaires (except SUS/NASA-TLX) not validated.<br><br>No long-term retention. Technical~za parameters may limit generalisability.

									teaching; qualitative suggestions: balance audio levels, more 3D, headphone use, adequate space.	
